# SARS-CoV-2 and innate immunity: the good, the bad, and the “goldilocks”

**DOI:** 10.1038/s41423-023-01104-y

**Published:** 2023-11-20

**Authors:** Benjamin L. Sievers, Mark T. K. Cheng, Kata Csiba, Bo Meng, Ravindra K. Gupta

**Affiliations:** 1 Department of Medicine, University of Cambridge, Cambridge, UK; 2 Cambridge Institute of Therapeutic Immunology & Infectious Disease (CITIID), Department of Medicine, University of Cambridge, Cambridge, UK

**Keywords:** SARS-CoV-2, Innate immune response, Interferon, Cytokines, Inflammation, Antiviral targets, Immune evasion, RIG-I-like receptors, Interferons

## Abstract

An ancient conflict between hosts and pathogens has driven the innate and adaptive arms of immunity. Knowledge about this interplay can not only help us identify biological mechanisms but also reveal pathogen vulnerabilities that can be leveraged therapeutically. The humoral response to SARS-CoV-2 infection has been the focus of intense research, and the role of the innate immune system has received significantly less attention. Here, we review current knowledge of the innate immune response to SARS-CoV-2 infection and the various means SARS-CoV-2 employs to evade innate defense systems. We also consider the role of innate immunity in SARS-CoV-2 vaccines and in the phenomenon of long COVID.

## Introduction

In late 2019, a novel respiratory disease named coronavirus disease 2019 (COVID-19) struck with ferocity, quickly becoming a global pandemic; this disease was later found to be caused by severe acute respiratory syndrome coronavirus 2 (SARS-CoV-2) [1–3]. There are multiple CoVs that infect humans, including common seasonal human coronaviruses (hCoVs), such as *Betacoronaviruses* HKU1 and OC43 and *Alphacoronaviruses* NL63 and 229E, and more uncommon CoVs, such as Middle East respiratory syndrome coronavirus (MERS-CoV) and severe acute respiratory syndrome coronavirus (SARS-CoV) [4–6]. SARS-CoV-2, consistent with other members of its genus, has a positive-sense, single-stranded RNA genome of ~30,000 nucleotides and produces approximately 30 proteins [7]. Starting from the 5′ terminus, the genes for the replicase and nonstructural proteins (ORF1a and ORF1ab) are present, followed by the spike (S), envelope (E), membrane (M), and nucleocapsid (N) proteins, with various intergenic accessory factors interspaced within this framework [8]. Two large polyproteins, ORF1a and ORF1ab (pp1a and pp1ab), are cleaved by viral proteases (PLpro and 3CLpro) into 16 nonstructural proteins (NSP) that predominantly constitute the RNA-dependent viral replicase, including four proteins that form the virion (S, M, N, E), and seven accessory proteins that play a pivotal role in manipulating cell biology and modulating viral pathogenesis (ORF3a, ORF6, ORF7a/b, ORF8, ORF9, and ORF10) [3, 7, 9]. COVID-19 manifests in a wide range of disease states, from mild nonspecific symptomology and even asymptomatic disease to moderate and severe illness requiring hospitalization [10–13]. Cytokine release syndrome (CRS) is a systemic condition seen in some severe COVID-19 cases that results from an overwhelming release of cytokines, which causes severe inflammation and induces acute respiratory distress syndrome (ARDS) and secondary hemophagocytic lymphohistiocytosis (sHLH) [14, 15]. To mitigate CRS, the innate immune system maintains endogenous feedback networks using cytokines such as IL-4, IL-10, IL-11, and IL-13 to drive anti-inflammatory phenotypes [16].

Innate immunity is an ancient system conserved from fish to mammals that provides the time necessary for the adaptive immune response to commence [17]. Various factors play a role in determining the severity of infection, and our innate immune response is central. Broadly, the role of the innate immune response to viruses is (1) to limit viral entry into a cell, block the translation of viral elements, the replication of the viral genome, and prevent egress of new infectious virions; (2) to identify and purge infected cells; and (3) to accelerate the development of a targeted adaptive immune response [18–20]. Activation of the inflammatory cascade, including the broad interferon response, plays a critical role in the clinical manifestation of SARS-CoV-2 [21, 22]. Various cell surface cytosolic and endosomal pattern recognition receptors (PRRs) in the cell surface and cytosol are activated once signaled by pathogen-associated molecular patterns (PAMPs), thereby triggering an inflammatory cascade and controlled cell death in affected cells [23–25]. As might be anticipated, excessive activation of this highly regulated system can lead to severe systemic inflammation [26, 27].

The release of IFN-I, along with various other inflammatory molecules following PRR activation, initiates antiviral defenses in neighboring cells in an attempt to limit further viral replication and spread [28–30]. While recent reports indicate that SARS-CoV-2 is sensitive to IFNs in vitro [31], even more so than its relative virus, SARS-CoV, the relationship between IFN release and disease presentation remains an area of interest. Innate immune cells, such as macrophages, dendritic cells, neutrophils, monocytes, and natural killer (NK) cells, modulate this response, as they are equipped with an array of PRRs capable of recognizing PAMPs and damage-associated molecular patterns (DAMPs) to initiate inflammatory pathways and foster these immune responses [32]. Although IFN-λ is primarily produced by epithelial cells rather than monocytes, IFN-λ appears to be more protective against infection and disease progression [33, 34].

Calibration of the type I interferon (IFN-I) response is critical to disease outcomes, as excessive or insufficient activation of IFN signaling can be life-threatening [35–37]. Mounting a robust IFN response at the onset of SARS-CoV-2 infection is of critical importance for developing a protective immune response, and suppression of IFN signaling contributes to severe COVID-19 disease states [14, 37]. Extended IFN production exacerbates disease progression by impeding the regeneration of lung epithelial cells [38, 39]. Evidence for the critical nature of the timing and magnitude of innate responses comes from data showing that although IFN-I can block infection in vitro, IFN-β may fail to provide therapeutic benefits if administered late in severe cases [31, 40–42]. Furthermore, the broad unbridled activity of the innate immune response and autoantibodies against IFN-I has been associated with severe COVID-19 [43–45].

Like many viruses, SARS-CoV-2 can evade the innate immune system through multiple strategies, including viral antagonism, avoidance of detection, and inflammatory response modulation (Table 1) [46–49]. Over- or under-activation of the innate immune response is detrimental in efforts to clear the infection; thus, a balanced response is needed as part of a “goldilocks” just-right scenario. In this review, we aim to summarize the innate immune response to SARS-CoV-2 infection with regard to disease modulation and immune system evasion and determine how we might manipulate this response for therapeutic benefit.

**Table 1:** Key SARS-CoV-2 proteins involved in counteracting host innate immune responses

SARS-CoV-2 protein	Mechanism of antagonism	Effect on the host’s innate immune response	References
NSP1	Inhibits IFN response through the depletion of key signaling factors	Reduces IFN production and signaling	(Kumar et al.) [186]
NSP3	Inhibits IFN-I production through the cleavage of IRF3	Reduces IFN production and signaling	(Moustaqil et al.) [262] (Alhammad et al.) [195] (Taha et al.) [192]
NSP13, NSP14, and NSP15	Inhibit nuclear localization of IRF3	IFN action and signaling antagonism	(Yuen et al.) [181] (Feng et al.) [263] (Fung et al.) [264]
ORF3a	Inhibits fusion of autophagosomes with lysosomes	Manipulates and antagonizes autophagy	(Zhang et al.) [162]
ORF3b	Inhibits the nuclear localization of IRF3	Acts as a potent interferon antagonist	(Konno et al.) [179]
ORF3c	Interacts with PGAM5 to induce caspase-3 cleavage of MAVS	Antagonizes IFN-β production, alters mitochondrial metabolism, blocks, autophagy	(Mozzi et al.) [163] (Stewart et al.) [174]
ORF6	Interacts with Nup98-Rae1 to blocks STAT1 and STAT2 nuclear translocation	Acts as a potent interferon antagonist	(Miorin et al.) [182]
ORF7a	Blocks SERINC5 incorporation into the virion and interferes with autophagosome acidification	Antagonizes autophagy and antiviral action	(Timilsina et al.) [86] (Hayn et al.) [48]
ORF8	Decreases nuclear translocation of IRF3 and mimics IL-17A	Antagonizes IFN production and downregulates MHC-I	(Rashid et.) [183] (Wu et al.) [209]
ORF9b	Interrupts the K63-linked ubiquitination of the interferon signaling modulator NEMO	Antagonize RIG-I-MAVS antiviral IFN-I response	(Wu et al.) [172]
ORF10	Triggers NIX-dependent (Nip3-like protein X) mitophagy leading to the degradation of MAVS	Degrades MAVs through mitophagy	(Li et al.) [173]

## Pattern recognition receptors (PRRs) of SARS-CoV-2

Consistent with most viral infections, one of the first steps initiated by the host’s innate immune response at the start of SARS-CoV-2 infection is the production and release of type I and type III interferons (IFN-I and IFN-III, respectively) [35, 50]. As SARS-CoV-2 infects a cell and viral replication commences, viral RNA is detected through a series of cellular PRRs, such as RIG-I-like receptors (RLRs), nucleotide-binding oligomerization domain (NOD)-like receptors (NLRs), and Toll-like receptors (TLRs), which are highly evolved cellular surveillance systems that specialize in detecting PAMPs associated with viruses or other pathogens [25, 51, 52].

Cytoplasmic RNA sensors, including TLR3 and RLRs such as MDA5 and LGP2, play a critical role in innate immunity by recognizing viral RNA [53]. In particular, RIG-I detects short dsRNA or 5′-pppRNA, and MDA5 detects long dsRNA. LGP2 also detects viral RNA and is known to be a positive regulator of MDA5- and RIG-I-mediated antiviral responses [54, 55]. Another pathway called 2′,5′-oligodenylate synthetase (OAS1)-ribonuclease L (RNase L) senses non-self dsRNA, cleaves ssRNA, and induces cell death [56]. In SARS-CoV-2 infections, OAS1 efficacy relies on the post-translational modification of prenylation and is significantly linked with severe COVID-19 prevention [57]. Prenylation is the covalent addition of a lipid near the C-terminus of a protein, this addition allows the protein to anchor to the cell membrane. Notably, this defense mechanism is absent in horseshoe bats, a possible reservoir for CoVs, indicating that horseshoe bats may have evolved to be optimal reservoirs of SARS-like CoVs [57]. Additionally, inborn errors of OAS-RNase L have been found to be associated with multisystem inflammatory syndrome in children (MIS-C), which involves the release of an excessive amount of inflammatory cytokines upon SARS-CoV-2 infection [58, 59]; this downstream effect highlights the importance of this pathway in IFN stimulation and disease outcome.

## Interferon inducible antiviral restriction factors

Interferon-induced transmembrane proteins (IFITMs) are proteins embedded in the lipid membrane of cells with the primary function of inhibiting fusion between the viral envelope and the host cell membrane [60, 61]. These IFITMs likely prevent fusion by changing membrane curvature and decreasing membrane fluidity; IFITMs have been shown to inhibit the entry of several viruses, including the Ebola virus, the influenza virus, and HIV-1 [62–64]. Three main IFITMs exhibit antiviral properties; IFITM1 is found on the plasma membrane, IFITM2 is found on late endosomes, and IFITM3 is found on early endosomes [65–67]. Interestingly, there have been conflicting findings on the role of IFITMs and SARS-CoV-2 infection. While IFITMs have widely been demonstrated to inhibit infection, in hCoV, OC43, IFITM2, and IFITM3 have been shown to increase viral entry into cells [68, 69]. Alternatively, in SARS-CoV-2 infections, early in vitro studies demonstrated that IFITM2 and IFITM3 but not IFITM1 sufficiently restricted viral entry into the cell [70, 71]. Additional studies showed that IFITM2 restricts viral entry more than IFITM3, likely because of the route of viral entry [72]. SARS-CoV-2 enters cells through membrane fusion and/or endocytosis mediated by the spike protein. The spike protein contains a receptor-binding domain (RBD) that interacts with the cellular receptor angiotensin-converting enzyme 2 (ACE2) and a polybasic cleavage site (PBCS) S1/S2 that is proteolytically cleaved by transmembrane serine protease 2 (TMPRSS2) and cellular cathepsin L [73, 74]. Depending on the status of spike cleavage and the relative abundance of TMPRSS2 on the plasma membrane, the virion enters through TMPRSS2-mediated membrane fusion or late endosomal entry via secondary cleavage [74–77]. Importantly, this cleavage is impacted by allostery; for example, the allostery between the NTD and the PBCS [73, 78, 79]. TMPRSS2 is found on lung and primary human airway epithelial cells and enables endosome-independent viral entry that avoids the antiviral actions of IFITM2 and IFITM3 [72, 80, 81]. This suggests that different SARS-CoV-2 variants have different sensitivities to the TMPRSS2-mediated entry pathway; thus, they may have different susceptibility to antiviral IFITMs [73, 82, 83]. The Delta variant appeared to have evolved toward plasma membrane fusion, and somewhat unexpectedly, the Omicron variant has evolved to primarily use endosomal entry; this may be the result of immune evasion and changes in spike cleavage efficiency [73, 84]. The Omicron variant had decreased cleavage efficiency in certain cell types and increased susceptibility to IFITM2 and IFITM3 [85], possibly contributing to the milder disease states seen with Omicron infections.

In addition to IFITMs that exert their antiviral function at membranous structures, SERINC5 is a cellular multipass transmembrane protein that is involved in lipid transport and biosynthesis and is most well known for its inhibition of human immunodeficiency virus (HIV) infection and for being a target of the HIV antagonist protein Nef [86–89]. SERINC5 is incorporated into budding virions and prevents viral entry by blocking virus‒cell fusion; SARS-CoV-2 ORF7a has been demonstrated to block the incorporation of SERINC5 in budding SARS-CoV-2 virions, thus antagonizing the antiviral action of SERINC5 [86, 87].

## cGAS-STING Axis

**Fig. 1:**
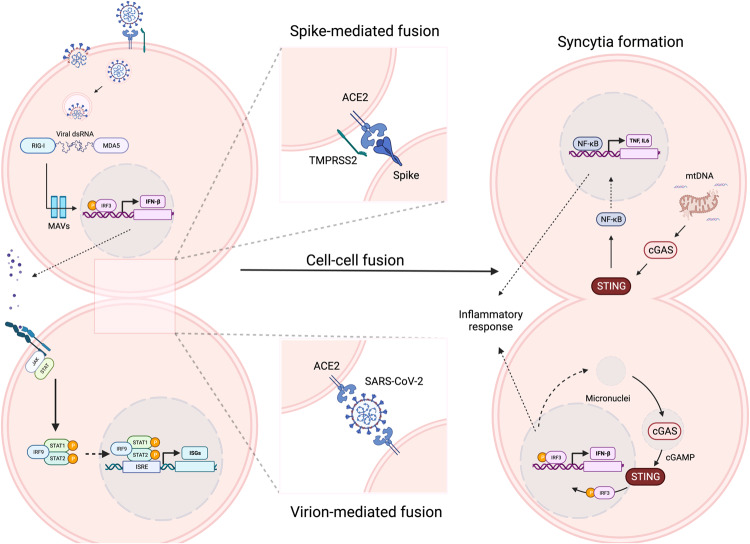
SARS-CoV-2 mediated cell-cell fusion and impact on cGAS-STING and IFN signaling

Cytoplasmic DNA sensors such as IFI16, AIM2, and cGAS play a critical role in identifying viral signatures and sensing cellular mitochondrial DNA (mtDNA) during RNA virus infection [36, 90, 91]. The Cyclic-GMP-AMP synthase STimulator of INterferon Genes (cGAS-STING) system [92] is broadly manipulated during SARS-CoV-2 infection; this system is involved in syncytial pneumocyte formation, cell-to-cell fusion, and reduced cytokine signaling [93–96]. Papain-like protease (PLpro), one of the virally encoded proteases whose activity may correlate with pathogenicity [97], also activates the antiviral IFN response by deubiquitinating K63-linked polyubiquitin chains of STING, therefore disrupting the cGAS-STING axis for the induction of IFNs and interferon-stimulated genes (ISGs) [98]. Cell‒cell fusion, where plasma membranes fuse to form multinuclei cells, is a well-documented phenomenon mediated by the SARS-CoV-2 spike protein and its receptor ACE2, a process triggered by spike cleavage to expose the fusion peptide. Syncytium formation has been observed in postmortem tissues associated with severe COVID-19 [99, 100] and extensively demonstrated in vitro [64, 73, 79, 101]. Transcriptomic analysis of spike-mediated fused cells showed that the IFN response was one of the most upregulated processes [94]. Further analysis showed that IFN is induced by cGAS, which was colocalized with large DNA aggregates due to nuclear membrane rupture, leading to the activation of IRF3. This IFN induction is dependent on spike cleavage and is increased in cells that overexpress host proteases, such as TMPRSS2, that are drug targets; furthermore, mutating the cleavage site completely abrogates IFN stimulation in fused cells [94]. Interestingly, damaged DNA released from the ruptured nucleus is not the only source for cGAS sensing, as SARS-CoV-2 infection also causes direct mtDNA damage [102]. However, as cGAS signaling requires cGAS-G3BP1 coassembly on dsDNA to form stress granules, the viral nucleocapsid restricts cGAS signaling by competitively binding G3BP1 to divert dsDNA into alternative liquid‒liquid phase-separation condensates (Fig. 1) [102].

## RNA editing-dependent antiviral innate responses

RNA viruses tend to have high mutation rates due to the high mutation rate of viral RNA-dependent RNA polymerase (RdRp), which limits their genome length as extensive mutations can lead to error catastrophe [103]. CoVs manage the largest genomes (~30 kb) among RNA viruses by encoding 3′-5′ exoribonucleases with proofreading activity that lower mutation rates up to 100-fold compared to that of other RNA viruses, such as influenza, HCV, and HIV [104–107]. RNA editing is a crucial innate immune response to endogenous and exogenous RNA sequences in both health and disease states; these modifications had an unintended impact on SARS-CoV-2 genome diversity [108–116]. In particular, one mRNA editing enzyme, apolipoprotein B (ApoB), which is part of the catalytic polypeptide-like (APOBEC) family that is further explored below, has been hypothesized to be increased in SARS-CoV-2-induced senescence alveolar type II (ATII) cells, which are a fertile source for generating hypermutated progeny [117, 118]. As new strains emerge, alterations in tissue tropism can redirect viral evolution. This is evident from the Omicron variant; the transition to upper respiratory tract replication occurred with a significant reduction of G > T in the mutational spectra compared to previous variants [119], which replicate both in the upper and lower respiratory tract.

In SARS-CoV-2 infection, cytoplasmic viral dsRNA from the transcription-replication complex is recognized as pathogenic non-self RNA by host antiviral proteins and sensors [57]. As the amount of adenosine deaminase that acts on RNA type I (ADAR1) and APOBEC3 is increased in the interferon innate immune antiviral response, modulation by the viral genome can suppress excessive immune reactions initiated by intracellular dsRNA sensors such as MDA5, OAS-RNase L, and protein kinase R (PKR) [120–122]. In addition, the recent discovery that dsRNA can trigger pyroptosis via NLRP1 sensing further suggests that the regulation of dsRNA signaling plays a role in SARS-CoV-2 pathogenesis [123].

### APOBEC

The APOBEC family includes restriction factors for a diverse range of viruses, including retroviruses, hepatitis C virus (HCV), herpesviruses, and foot and mouth disease virus [124, 125]. The key members of this family that are capable of single-stranded RNA deamination are APOBEC1, APOBEC3A, and APOBEC3G [126]. Aberration of viral replication is achieved by performing lethal editing via cytidine deaminase activity in a sequence- and structure-dependent context [127]. While the exoribonuclease grants CoVs a level of “immunity” against APOBEC editing-induced mutagenesis, studies of global SARS-CoV-2 consensus sequences deposited in Global Initiative on Sharing All Influenza Data (GISAID) revealed signatures of APOBEC-mediated C-to-U transition. This change is the most abundant SARS-CoV-2 mutation, accounting for up to 46% of nucleotide substitutions [103, 114, 128]. The frequency of this mutation is supported by the asymmetric abundance in C-to-U transitions in specific dinucleotide contexts (TC, AC, or CC) and the high non-synonymous mutation to synonymous mutation ratio, which suggests non-neutral evolution driven by additional mutational mechanisms beyond random changes seen in genetic drift [109, 129–132].

### ADAR

Another family of RNA editing enzymes is the ADAR gene family. In contrast to the APOBEC family, ADAR recognizes dsRNA, a common structure found in replication complexes and secondary RNA structures [133]. Within the ADAR family, only ADAR1, and more specifically, ADAR1 isoform p150, is interferon-inducible and shuttles between the nucleus and cytoplasm [134]. ADAR has the important function of regulating cytoplasmic innate immunity by deaminating adenosine to inosines. This destabilizes the dsRNA structure between complementary strands or secondary RNA structures, such as hairpin loops formed by retrotransposons known as Alu repeats [135]. Failure to eliminate endogenous dsRNA leads to a constitutive antiviral response and inflammation, as seen in the autoimmune disorder Aicardi-Goutiéres Syndrome [135, 136].

The interest in ADAR1 sparked after the acquisition of the A23403G mutation that led to the spike D614G amino acid change [137]. While there is evidence of upregulated expression and RNA‒protein interactions involving ADAR1, such as interactions with APOBEC3A, IFN-responsive SARS-CoV-2-infected human cells and RNA-seq of patient bronchoalveolar lavage samples showed A-to-G/T-to-C biases; there has been limited emergence of ADAR1-related mutations in in vitro models and the general population [8, 110, 138–140]. Nevertheless, deep sequencing analysis showed ADAR1 mutation signatures in minor viral populations that were inversely correlated with viral load, mortality, and incidence, suggesting that ADAR1 mutations may be significant [141].

### ZAP

Last, zinc-finger antiviral protein (ZAP, also known as poly(ADP-ribose) polymerase 13 (PARP13)) binds to CG dinucleotides and recruits the cofactors KHNYN and TRIM25 to degrade viral RNA [142–146]. The antiviral activity of ZAP can be potentiated by a cellular polynucleotide poly(ADP-ribose) [147]. ZAP expression is upregulated in the SARS-CoV-2 innate immune antiviral response [148], and thus, the SARS-CoV-2 virus has shown reduced CG content since its emergence; this change occurred as an adaptation to circulation in human hosts [149].

## Programmed cell death

Autophagy is an evolutionarily conserved cellular process that can flip between beneficial and harmful actions amidst an active viral infection [150, 151]. Upon SARS-CoV-2 infection, abundant cellular machinery and organelles serve as sanctuaries for viral replication, thereby enabling extended replication and continuous infection. Activation of well-timed and appropriate autophagy mounts a counterattack on virus-producing compartments by initiating cell death and degrading the viral particles within infected cell [152–154]. However, there is a molecular arms race between viruses and humans, as some viruses, such as poliovirus, HIV, HCV, and SARS-CoV-2, can manipulate autophagy for their benefit [153, 155, 156]. Autophagy requires a delicate balance, and perturbations, such as those induced by viral antagonistic proteins, can tilt the scale in favor of viral success. However, inappropriate or excessive autophagy can contribute to cellular damage and systemic inflammation, disrupt normal cell function and exacerbate cytokine responses often associated with severe COVID-19 [48, 153].

In SARS-CoV-2, the NSP15, ORF3a, ORF3c, ORF7a, ORF10, E, and M proteins have been reported to manipulate and antagonize autophagy [157–160], with the ORF3a and ORF7a proteins appearing to be dominant [48]. Although these proteins broadly prevent autophagy, they do so in a myriad of mechanisms. ORF3a prevents autophagy by inhibiting the fusion of autophagosomes with lysosomes by sequestering the homotypic fusion and protein sorting (HOPS) component VPS39, which prevents assembly of the SNARE complex [161, 162]. On the other hand, ORF7a was shown to prevent autophagy by reducing the acidity of the lysosome by increasing the pH [48]. This is complemented by ORF3c hyperactivation of oxidative phosphorylation, which induces the overproduction of ROS and compromises lysosomal acidity and autophagy [163].

Pyroptosis, a proinflammatory form of programmed cell death utilized in the innate immune response, was observed in the lung tissues of patients with severe COVID-19 [164]. Using transcriptome analysis, NSP6 was demonstrated to trigger NLRP3-dependent pyroptosis by targeting ATP6AP1, a vacuolar ATPase proton pump component [165]. Interestingly, pyroptosis observed in FcγR-mediated SARS-CoV-2 infection of monocytes and macrophages led to abortive replication and systemic inflammation, which contributed to COVID-19 severity [166, 167]. However, pyroptosis can be blocked through N binding to the Gasdermin D (GSDMD) linker region, which prevents GSDMD cleavage that is needed for the initiation of pyroptosis [168, 169]. This finding indicates that a GSDMD inhibitor could be potential approach to counter excessive inflammation in severe COVID-19.

## Viral antagonism of cytokine and IFN signaling

**Fig. 2:**
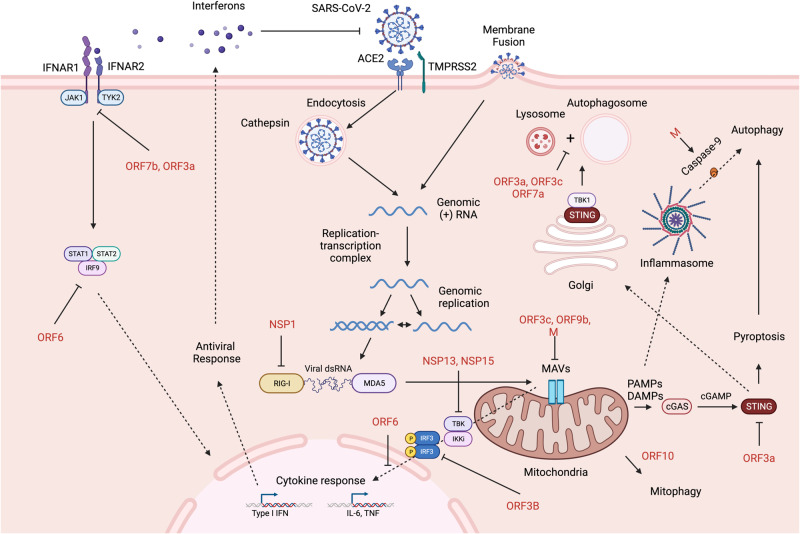
SARS-CoV-2 cell entry and early life cycle, innate immune pathways, and virus-encoded antagonists

Upon the detection of SARS-CoV-2 viral RNA through one of the cytoplasmic RNA sensors, downstream activation via mitochondrial anti-viral signaling protein (MAVs) induces the phosphorylation of interferon regulatory factor (IRF) and nuclear translocation (Fig. 2). This cascade event can lead to the activation of immune genes, including IFN-I and IFN-III, and an antiviral response. In SARS-CoV-2 infections, a deficiency in MDA5, MAVS, or RIG-I can lead to enhanced viral replication [170]. The SARS-CoV-2 glycoprotein M was demonstrated to impair MAVS aggregation and the further recruitment of TNF receptor associated Factor 3 (TRAF3), TANK binding kinase 1 (TBK1), and IRF3 sufficiently attenuated the innate antiviral response [171]. Through a different mechanism, SARS-CoV-2 ORF9b was also shown to antagonize the RIG-I-MAVS antiviral IFN-I response by preventing the K63-linked ubiquitination of the interferon signaling modulator NEMO [172]. Alternatively, ORF10 was also shown to suppress the antiviral innate immune response by triggering NIX-dependent (Nip3-like protein X) mitophagy, which led to the degradation of MAVS [173]. Finally, ORF3c, a recently discovered 41-aa peptide product of leaky ribosomal scanning (+1 reading frame) entirely nested within the ORF3a sgRNA, localizes and interacts with both MAVS and mitochondrial protein phosphoglycerate mutant family member 5 (PGAM5) to induce caspase-3-mediated cleavage of MAVS [174]. RIG-I may also have noncanonical action against SARS-CoV-2 by competitively inhibiting RdRp via binding to the SARS-CoV-2 3′ UTR without triggering ATPase and thus downstream activity [175].

*Betacoronavirus* CoVs, including SARS-CoV and MERS-CoV, use several strategies also used by SARS-CoV-2 to avoid innate immune detection. MERS-CoV, consistent with the IFN antagonism employed by SARS-CoV-2, regulates IRF3 nuclear trafficking with ORF4a, ORF4b, ORF5, and the membrane protein [176]. Similarly, SARS-CoV-2 ORF6 was shown to interfere less efficiently with interferon signaling than SARS-CoV ORF6 [177]. Collectively, despite marked differences in entry mechanisms, transmissibility, and pathogenicity among coronaviruses, these viruses have evolutionarily converged strategies for evading our immune system [89].

IFN responses in SARS-CoV-2 infections appear to remain weak overall, potentially indicating efficient antiviral evasion/antagonism of PRRs [37, 178–180]. The NSP13, NSP14, NSP15, ORF8, ORF3b, and ORF6 proteins act as potent interferon antagonists and contribute to suppression of the primary interferon response [179–181]. NSP14 targets the IFN-I receptor (IFNAR1) for lysosomal degradation [48]. Alternatively, ORF6 inhibits the IFN-I response by blocking STAT, and ORF8 inhibits IFN production by reducing the nuclear translocation of IRF3 [180, 182, 183]. Another study demonstrated that the D61L mutation in ORF6 is responsible for the decrease in IFN-β secretion in vitro [184], which might account for the reduced clinical severity seen in Omicron induced diseases [185]. Likewise, more recently, the SARS-CoV-2 ORF3b protein has also been demonstrated to be a potent interferon antagonist; this protein suppresses IFN-I induction more efficiently than its ORF3b ortholog in SARS-CoV [179].

Some SARS-CoV-2 proteins play multiple roles in the manipulation of the IFN response, such as NSP1, which prevents IFN induction through the blockage of IRF3 phosphorylation and the depletion of the IFN-I signaling components, TYK2, and STAT2 [186]. Experimental evidence also demonstrated that NSP3 antagonizes IFN-I production through the cleavage of ISG15 from IRF3, which is associated with MDA5 signaling [187, 188]. NSP3 contains a pancoronavirus-conserved macrodomain (Macro1) that is essential to pathogenesis [189–192], although slight differences in residues define their specificity [193]. Depending on the ADP-ribosylation target, interferon-responsive poly(ADP-ribose) polymerases can act as positive and negative regulators of the innate antiviral response [194]. SARS-CoV-2 virus bearing Macro1 deletion of a single site in its catalytic domain increased the levels of IFNs and ISGs both in cell lines and mice, indicating that Macro1 is an antagonist of IFNs and explaining the attenuated pathology in mice [192, 195]. Macro1 reverses global PARP9/DTX3L ADP-ribosylation upregulation of IFN-I and -II expression without preventing the induction of ISGs. As PARP9/DTX3L targets host histones to promote IFN signaling and viral proteins for degradation [196], ADP-ribosylation likely plays an undiscovered role in SARS-CoV-2 infection.

NSP6 was shown to bind TBK1, which also suppresses IRF3 phosphorylation [197]. In addition to their primary role in facilitating RdRp activity, NSP7 and NSP8 have also been demonstrated to suppress IFN-I responses, with NSP8 binding to MDA5 CARD to block K63-linked polyubiquitination, a key step involved in the regulation of the innate immune response [198, 199].

Among all proteins, SARS-CoV-2 ORF8 is the most hypervariable in SARS-CoV-2, following the spike protein, and shares the least homology among earlier major human coronaviruses [200]. At the sequence level, ORF8 shares greater homology with bat and pangolin coronaviruses than SARS-CoV (~90% sequence homology with BAT-SL-CoVZC45 vs. ~30% with SARS-CoV) [201, 202]. In addition, SARS-CoV gained a 29-nt deletion, splitting into ORF8a and ORF8b [203]. SARS-CoV-2 ORF8 has de novo functions not seen in SARS-CoV or MERS-CoV. Although paralogs that share the immunoglobulin (Ig)-fold-like structure can be found, such as ORF7b, SARS-CoV-2 ORF8 is unique in its ability to dimerize and has lost its C-terminal transmembrane domain, allowing secretion from infected cells [200, 204, 205].

Interestingly, milder infection was observed in 39 Singaporean patients infected by a virus with a 382-nucleotide deletion (∆382), which truncated ORF7b and eliminated the ORF8 leader transcriptional regulatory sequence (TRS-L) [206]. ORF8 ablation either by truncation or TRS deoptimization has been shown to be under positive selection and has increased transmissibility in certain lineages, including the B.1.1.7/Alpha (Q27*), BA.5 (C27889T), XBC (G27890T), and XBB.1 (G8*) sublineages, and reached a 80% global prevalence [207]. Although there is a de facto loss of ORF8 function, more transmissible variants often have mutations in the spike protein and other regions, possibly explaining the increased pathogenicity of the Alpha variant despite ORF8 truncation [208].

ORF8 is an interferon antagonist that disrupts epigenetic-related posttranslational modifications (PTMs) of histones; this disruption prevents detection by MHC-I, abolishes interferon production and signaling, and mimicks cytokine signaling [209, 210]. Wuhan-1 and VoCs of SARS-CoV-2 ORF8 possess an ARKSAP motif, which has not been previously observed in SARS-CoV but is present in BAT-SL-CoVZC45. The ARKS motif is a PTM site critical to histone H3 at lysine 9 and lysine 27 (H3K9 and H3K27) [210]. Kee and colleagues demonstrated that the histone mimic ORF8 disrupts chromatin accessibility and results in significant differences in gene expression [210].

Dimerized ORF8 also mediates MHC-I degradation through autophagy pathways, thereby providing protection against CTLs [202, 211]. ORF8 also inhibits global protein synthesis by interfering with the ER-Golgi process and induces ER stress by activating the transcription factor (ATF6) and inositol-requiring enzyme 1 (IRE1) pathways [212, 213]. The expression of antiviral interferon-stimulated genes is further downregulated by inhibiting the phosphorylation of IRF3 and thus limiting nuclear translocation [213, 214].

## SARS-CoV-2 evolution, variants of concern, and increased antagonism

SARS-CoV-2 VoC has emerged sequentially from the ancestral B.1 lineage throughout the pandemic and often harbors mutations in key viral proteins that modulate immune antagonism and evasion [215, 216]. Although infectivity and antibody evasion are the two primary factors that drive SARS-CoV-2 evolution [217], it has become increasingly clear that IFN resistance may also play a critical role in shaping the trajectory of SARS-CoV-2 [216, 218].

The Alpha variant was shown to suppress innate immune responses in vitro to a greater extent than first-wave isolates. Furthermore, there was an increase in the subgenomic RNA and protein levels of ORF9b, ORF6, and N, which are known to antagonize the innate immune response [47]. Interestingly, the Alpha variant had reduced secretion of IFN-β due to lower amounts of dsRNA intermediates sensed by cells [47, 219]. These innate immune evasion strategies may contribute to the increased transmissibility and enhanced innate immune evasion seen with the alpha variant. While the Alpha variant demonstrated high transmissibility in the human population [220], this level of transmissibility is yet to be fully established in vitro [221], with one study showing a spike-dependent replication advantage in low ACE2-expressing bronchial cell lines compared with the ancestral B.1 [222]. Interestingly, the Alpha variant contains a P681H mutation within PBCS in the spike protein that enables IFITM escape and increases IFN-I resistance [223].

Like IFITM, which is a broad ISG that can restrict viral entry, interferon-inducible restriction factor guanylate-binding proteins (GBPs) have been demonstrated to inhibit furin-mediated processing of viral envelope proteins, including SARS-CoV-2 [85]. Notably, in 2020, the evolution of a D614G substitution on the spike protein of SARS-CoV-2 allowed the mutated virus to escape GBP restriction [137]. Early lineage isolates of Wuhan-Hu-1 and BetaCoV/Australia/VIC01/2020 (VIC) remain susceptible to GBP2 and GBP5, but VoC such as Alpha and Delta escaped GBP2/5 restriction [85]. Interestingly, the Omicron variant remains sensitive to GBP2/5 and endosomal IFITMs [85], likely because of the alternate cell entry pathways associated with different selective pressures and spike mutations [73, 224, 225].

Sensitivity to interferon also varied greatly among patients with VoC. The Omicron variant [226] showed reduced antagonism of the host interferon response compared to the Delta variant [227, 228]; however, Omicron maintained resistance to interferon treatments, in contrast with Delta [229, 230]. Likewise, through integrative computational analyses, the Alpha, Beta, Gamma, and Delta VoC suppressed ISGs, yet the Omicron variant did not [231]. Overall, there is increased interferon resistance among VoC lineages when compared with ancestral isolates, suggesting that innate immune evasion may play a critical role in driving and shaping SARS-CoV-2 evolution [218].

## The innate immune response to vaccines

The goal of vaccination is to sufficiently prime the immune system against an infectious agent to prevent future disease. An effective vaccination program has proven to be critical in the fight against the COVID-19 pandemic, with the following four main methods leading the charge: mRNA vaccines, viral vector vaccines, inactivated vaccines, and protein subunit vaccines [232–234]. Since the start of the pandemic, primary T and B-cell responses following vaccination have been abundantly characterized, yet the role of the innate immune system in protection following vaccination has received less attention.

Following BNT162b1 vaccination, there is preferential stimulation of classical (CD14^bright^ CD16^−^) and intermediate (CD14^dim^/CD16^dim^) monocytes but a reduction in nonclassical (CD14^dim^/CD16^bright^) monocytes compared to their baseline levels [235]. Even 6 months after the first booster, the percentage of nonclassical monocytes was reduced compared to baseline levels. These vaccine-induced changes shown in the monocyte subpopulations also highlight activation of the protective innate immune response by vaccination, as classical monocytes are critical for the initial inflammatory response, whereas nonclassical monocytes have an anti-inflammatory role and respond poorly to TLR stimulation [235, 236].

Interestingly, BNT162b1 vaccine-induced antibodies have been shown to activate CD107a by NK cells at a greater rate than antibodies generated by natural infection [237]. Despite the diminished CD16 expression, the increased NK cell activity may be explained by the differences in the activation of stimulatory and inhibitory receptors on NK cells following BNT162b1 vaccination. Stimulatory 2DS2-expressing NK cells were significantly augmented after 3 doses of vaccine compared to the baseline levels, while the opposite effect was observed in terms of inhibitory KIR receptor expression. The amount of ILT-2-expressing NK cells, in particular, was significantly reduced [235].

Not only have mRNA-based vaccines been observed to provide enhanced innate immune protection against SARS-CoV-2 infection through the differential activation of innate immune cells, but recent data have also shown an increased innate immune response following dose 2 compared to that following dose 1 [238]. The frequency of intermediate monocytes (CD14^+^ and CD16^+^) increased significantly two days after vaccination dose 1 and was substantially higher two days after dose 2. In addition, there were increased levels of pSTAT3 and pSTAT1 in multiple cell types on day one after dose 2, relative to that on day one after dose 1 vaccination. This suggests that BNT162b2 vaccination induced a more substantial innate immune response after dose 2 [238]. These findings are particularly revealing when considering that one of the hallmarks of SARS-CoV-2 infection is impaired IFN-I and III production and responses [239].

Enhancement of innate immune responses was also observed post-BNT162b2 vaccination in individuals previously vaccinated with two doses of the AZD1222 course [240]; Ferreira et al. observed a greater increase in interferon alpha and gamma mRNA signatures and lymphocyte costimulatory signatures by scRNA-seq one month following the mRNA booster dose in comparison to a similar time point post dose 2 of AZD1222 [240]. These innate responses appeared somewhat blunted in elderly individuals [240], and this may underlie reduced spike-specific B and T-cell responses in this vulnerable group [240, 241]. Similarly, reduced germinal center function has been observed in aged mice following vaccination [242], likely due to the microenvironment rather than intrinsic defects [242, 243]. Consistent with the critical role of innate signaling in driving robust vaccine responses, TLR4 can boost germinal center responses to immunization in aged mice by promoting innate immune signaling [244].

Neutralizing antibody is known to predict immune protection [245]. Although showing a lower level of neutralizing antibody in comparison to mRNA vaccines [73, 246–249], inactivated vaccines provided similar protection from this disease at three doses [250]. Unlike mRNA- or vector-based vaccines where the spike protein is the only immunogen, inactivated vaccines produce broader immune responses due to the presence of other structural proteins (M, N, and E). Therefore, it is not too surprising that although the inactivated vaccine elicited a lower magnitude spike-specific T-cell response, it produced a broader multiprotein-specific T-cell response with M, N, and spike-specific T cells, which can more efficiently target highly mutated variants, such as Omicron, compared with the response generated from mRNA vaccines [250]. Moreover, a higher frequency of HLA-DR^hi^ classical and nonclassical monocytes was found to be positively correlated with asymptomatic Omicron breakthrough infection in patients who had three doses of inactivated vaccines [251]. It was suggested that a booster vaccine could train immunity by priming monocytic activation and differentiation rather than suppressed monocytes upon SARS-CoV-2 infection.

## Long COVID-19 and the innate immune response

Long COVID-19, also known as the postacute sequelae of COVID-19 (PASC), is a chronic multisystemic condition with a wide range of symptoms that can occur following the resolution of SARS-CoV-2 infection. While substantial progress has been made in characterizing the illness and identifying pathophysiological changes, the etiology of this condition has yet to be revealed, and dysregulation of the innate immune response may play a critical role in the manifestation of associated symptoms.

Early studies reported that some individuals with long COVID had highly activated innate immune cells with elevated expression of IFN-I and IFN-III persisting 8 months post infection [252]. Likewise, other studies have noted elevated levels of cytokines, such as TNF, IP10, IL-1β, and IL-6, in long COVID individuals [253, 254]. These elevated levels of interferons and other proinflammatory cytokines may contribute to the chronic inflammation and immune dysfunction seen in individuals with long COVID.

Various forms of mitochondrial dysfunction were also identified in individuals with long COVID, such as altered fatty acid metabolism [255] and the loss of mitochondrial membrane potential [256]. Abnormal levels of mitochondrial proteins, along with abnormal levels of the S and N proteins, were also found in the central nervous system [257]. Reactivation of latent infections, including human herpesvirus (HHV)-6 and Epstein Bar virus (EBV), has also been identified in individuals with long COVID; this reactivation can induce mitochondrial fragmentation [254, 258–261]. Conceivably, mitochondrial dysfunction and disruption seen in individuals with long COVID-19 may contribute to increased activation of the MAV-IFN signaling pathway, which would inform disease presentation.

## Conclusions and perspectives

The host‒virus arms race is complex and involves both the innate and adaptive arms of the immune system. For SARS-CoV-2, the emergence of VoC has allowed us to witness how evolution is used to evade immunity in an unprecedented way. In the future, we could develop pharmacological approaches to exploit or harness our knowledge regarding innate immunity and viral evasion. For example, when a virus encodes a protein such as ORF8 that mimics a human protein, we could develop a specific antagonist of the viral protein. Similarly, pharmacological blockade of ORF3a and ORF7a, which would eliminate their contribution to the persistence of virally infected cells, might tip the balance toward expedited viral clearance. Alternatively, manipulation of PRRs or the cGAS-STING pathway might reduce systemic inflammation and improve COVID-19 outcomes. In particular, future work needs to consider the delicate balance between early protective innate responses and delayed chronic inflammatory responses and how humans and other mammalian hosts, such as bats, find the so-called “goldilocks” zone. Specific bat adaptations, such as mutations in ASC that dampen inflammasome activity and allow the asymptomatic carriage of viruses, may shed light on the drivers of pathological inflammation in humans that are amenable to pharmacological manipulation.
